# Dr. Phineas H. Strong

**Published:** 1890-03

**Authors:** 


### Obituary.

DR. PHINEAS H. STRONG, one of the most respected physicians of Buffalo, died at his home, in this city, on Monday, February 10, 1890, aged seventy-two years. Dr. Strong was a native of Vermont, but came to Buffalo in 1846, where he has since resided, and during these forty-four years has taken an active part in the medical affairs of this region. He has held nearly all the offices in the public medical service and in medical societies here, and was in earlier years a frequent contributor to the columns of the JOURNAL. He was appointed Professor of Medicine in Howard University, at Washington, D. C., soon after its organization, which chair he held for three years.

Dr. Strong was the embodiment of all that is graceful, courtly, and sympathetic in professional walks, and died respected by his fellow-citizens as well as his confrères.

At a special meeting of the Medical Society of Erie County, held Tuesday evening, February 11, 1890, the president, Dr. G. W. McPherson, appointed the following named gentlemen a committee to prepare a memorial of the late Dr. Strong, viz.: Drs. F. W. Bartlett, A. A. Hubbell, and John Hauenstein. They subsequently reported as follows:

*Resolved,* That in the death of the late P. H. Strong, the Medical Society of Erie County has lost one of its oldest, most devoted, and honorable members, one who exemplified in his professional and social life the highest qualities of a man and physician.

*Resolved,* That by his steadfast devotion to the advancement and education of the Society, he deserves the highest encomiums we can pay to his memory.

*Resolved,* That as a further tribute to his work and memory we will attend his funeral in a body.

Remarks upon the life and character of Dr. Strong were made by Drs. Howe, Hayd, Samo, W. W. Potter, and others, after which the memorial was adopted by a rising vote.

The funeral was held Thursday afternoon, February 13th, at the North Presbyterian Church, and was largely attended. The pall-bearers were Dr. George N. Burwell, Dr. Thos. Lothrop, Dr. James B. Samo, Dr. S. S. Green, Mr. Pascal P. Pratt, and Mr. John Otto.

The carriers appointed by the Society, at the request of the family, were: Drs. Wm. H. Thornton, DeLancey Rochester, E. H. Norton, Herman Hayd, James S. Porter, and W. H. Bergtold.

